# Changes in Benthos Associated with Mussel (*Mytilus edulis* L.) Farms on the West-Coast of Scotland

**DOI:** 10.1371/journal.pone.0068313

**Published:** 2013-07-12

**Authors:** Thomas A. Wilding, Thomas D. Nickell

**Affiliations:** Scottish Association for Marine Science, Scottish Marine Institute, Oban, Argyll, United Kingdom; University of Southampton, United Kingdom

## Abstract

Aquaculture, as a means of food production, is growing rapidly in response to an increasing demand for protein and the over-exploitation of wild fisheries. This expansion includes mussels (family Mytilidae) where production currently stands at 1.5 million tonnes per annum. Mussel culture is frequently perceived as having little environmental impact yet mussel biodeposits and shell debris accumulate around the production site and are linked to changes in the benthos. To assess the extent and nature of changes in benthos associated with mussel farming grab and video sampling around seven mussel farms was conducted. Grab samples were analysed for macrofauna and shell-hash content whilst starfish were counted and the shell-hash cover estimated from video imaging. Shell-hash was patchily distributed and occasionally dominated sediments (maximum of 2116 g per 0.1 m^2^ grab). Mean shell-hash content decreased rapidly at distances >5 m from the line and, over the distance 1–64 m, decreased by three orders of magnitude. The presence of shell-hash and the distance-from-line influenced macrofaunal assemblages but this effect differed between sites. There was no evidence that mussel farming was associated with changes in macrobenthic diversity, species count or feeding strategy. However, total macrofaunal count was estimated to be 2.5 times higher in close proximity to the lines, compared with 64 m distance, and there was evidence that this effect was conditional on the presence of shell-hash. Starfish density varied considerably between sites but, overall, they were approximately 10 times as abundant close to the mussel-lines compared with 64 m distance. There was no evidence that starfish were more abundant in the presence of shell-hash visible on the sediment surface. In terms of farm-scale benthic impacts these data suggest that mussel farming is a relatively benign way of producing food, compared with intensive fish-farming, in similar environments.

## Introduction

Aquaculture is growing rapidly in response to an increasing demand for protein and the over-exploitation of wild fisheries [1]. Sectors of the aquaculture industry that are expanding include predatory fish (e.g. salmon) but such species are reliant on protein- and oil-based feeds that are derived from increasingly limited marine and/or terrestrial sources [2]. Filter-feeding bivalves do not require human intervention in terms of feeding and their culture offers a potentially low-impact (see below) and sustainable means of producing high quality protein for an expanding human population [3] and/or a high value food product that brings economic benefits to the coastal communities where they are grown (e.g. [4]). Global mussel production, which has doubled in the last decade, currently exceeds 1.5 million tonnes per annum (FAO Statistics).

Mussels are farmed by stocking suitable sites with juveniles and allowing them to grow for a period prior to harvest. Sites can consist of areas of seabed (reviewed in [5]) or the water column. Mussels can be supported in the water column by wooden poles [6], [7], underneath rafts [8]–[10] or, as reported here, suspended on lines strung between floats. Mussels feed by pumping water through specially adapted gills that act as filters and trap particulate material [11]. Trapped particles are wrapped in mucus and either ingested or ejected as pseudofaeces. True faeces and uningested pseudofaeces (collectively known as biodeposits) are dispersed within and around the farm according to currents and water depth and, to some extent, accumulate on the seabed [11] together with living shells lost from the farm and associated infrastructure (see below).

Mussels are very effective at removing particulate material from seawater and redirecting it to the seabed and, consequently, were considered ecosystem engineers by Newell (2004) [11]. The removal of particulate material will have consequences at several levels, including the supporting water body, by potentially reducing food availability and water flow to natural populations [12]–[14], changing water column phytoplankton assemblage structure [15] and altering benthic nutrient recycling and benthic-pelagic coupling [11]. The macrobenthic infaunal consequences of the accumulation of organic material on the seabed has been extensively studied and occurs on a continuum from undetectable to a system that is azoic in respect of macrobenthos (described below). The macrobenthic response to mild organic enrichment is frequently an increase in abundance and diversity that is associated with the increased food supply [16]. As the organic loading increases the sediment becomes increasingly deficient in oxygen and enriched with sulphides (the metabolic end-product where sulphate is used as a respiratory terminal electron sink) [16]. At this stage longer-lived, larger macrobenthos are usually absent and the macrobenthic assemblage is characterised by superabundant, sulphide-tolerant opportunistic species and is of low diversity. As the organic loading increases further even these specially adapted species cannot tolerate the conditions and the sediment becomes azoic [16], [17].

Mussel farms are also associated with another biodeposit, that of living shells. Living shells are lost from their supports to the seabed through intra-specific competition, storm-damage, indirectly via bird predation or deliberately through infrastructure cleaning [18], [19]. These living shells not only attract predators (see below) but their remains, in various states of degradation (termed shell-hash), have the capacity to enhance the accumulation of biodeposits, and consequent changes in benthos, through increasing the benthic boundary layer thickness [20] in the same way as recorded for maerl [21].

Macrobenthic infauna are routinely assessed in relation to fish-farm impact monitoring [22], [23] and a similar approach has also been applied to mussel-farms (reviewed in [24]). The normal sampling approach is to obtain sediment samples, using a grab, and to pass this material through a sieve (normally with a 1 mm screen) and identify and enumerate the retained macrofauna. Varying analytical approaches can be adopted to assess the significance of site-dependent differences in the species-by-site matrix. These include sensitive multivariate techniques [25], [26] or, following data summarisation (e.g. to site-specific Shannon diversity index or a total species count), univariate modelling. Univariate models have the advantage of allowing an estimate of the parameter describing the relationship between the response and the predictors.

A high degree of location-specific variability in the macrofaunal response to mussel farm proximity has been recorded with both increases [27] and decreases [28]–[30] in abundance and biodiversity (increased [27], decreased [31]–[33]). A high degree of farm specific variability was also reported by Wilding [34] (this paper’s companion) in relation to redox, a proxy for sediment oxygenation and potential driver of macrobenthic assemblage change (see above). Wilding [34] showed that redox was lower at the farm periphery and that it increased, non-linearly, with distance. Whilst there are numerous macrobenthic studies (see above) comparing farm-proximal and farm-distant (‘control’) stations there is currently very little information on the functional relationship between farm distance and the degree and/or nature of farm-associated change (‘footprint size’). Furthermore, whilst there are some reports of increased habitat heterogeneity and biodiversity associated with the addition of shell-hash (a hard substratum) to an otherwise muddy habitat (reviewed in [24]) there are no reported assessments of the impact that shell-hash has on infaunal communities.

Compared to the macrobenthos much less is known about the impacts of activities, such as aquaculture, on the megabenthos, primarily because this group is logistically difficult to monitor [35]. The loss of living shells from mussel farms (see above) attracts predators and/or scavengers such as crabs and starfish [36], [37] with Inglis [19] reporting starfish densities up to 39 times higher under a mussel farm compared with farm-distant stations. However, other research (reviewed and tabulated in [24]) has found no significant changes in megabenthos in relation to mussel farms but this may simply reflect the challenges with respect to monitoring megabenthos (i.e. low experimental power [35]). To date there has been no research into the functional relationship between farm-distance and the megabenthos (rather than comparing near-farm and farm-distant stations) or how this varies between sites [24].

Whilst the physical presence and biodeposit-input from mussel farms means that they will alter both the macro- and megabenthic assemblages around them this has been rarely quantified [24]. Given our current understanding of mussel farms, and the regulatory interest in establishing their footprint size, the research reported here aims to quantify the functional relationship between impact indicators and farm-distance (and covariables) in order to explain and describe the nature and spatial extent of detectable farm-induced changes.

This research follows on from that presented in Wilding [34]. The goals of this research were to (i) determine the extent of the shell-hash field around mussel lines (ii) establish how the macrobenthos (consisting of assemblage structure, total count, diversity, richness and ITI) changes in relation to mussel farm distance and how/if this is linked to the shell-hash content of sediments and (iii) determine the relationship between mussel farm distance and the abundance of scavenger/predators and demonstrate if this is associated with the amount of visible (surface) shell-hash. These objectives were achieved.

## Methods

### Site Selection

In Scotland, a single mussel farm consists of one or more sites hosting a series of floats or rafts from which mussels are suspended on ‘droppers’ that hang into the water column. The lines are commonly in multiples of 220 m (a standard rope length). The mussels are normally deployed in an array running parallel to the shore or across bays. In order to examine changes in the benthic environment around mussel farms, sites were selected that were within 50 km of the laboratory (for logistical reasons) and where the outer line hosted a standing crop of mussels (mussels of harvestable age i.e. >two years old) that was adjacent to an area of sediment (as opposed to a rocky substratum) over/in which sampling could occur. The presence of the crop was clearly indicated by the height of the line-float in the water and was corroborated by slightly raising one or more droppers and/or visually using the drop-down-video (see below).

Inference to all farms within the sampled population was desired and, therefore, mixed modelling was the appropriate statistical framework for data analysis [35]. Assigning ‘Site’ as a random factor meant that direct comparisons between lochs, or sites within lochs, were not appropriate but that site-specific factors not accounted for in the measured co-variables would be accounted for (but not distinguished) in the random term [35]. The focus of the research reported here is on patterns in response variables as a function of distance from farm, not whether impact-metric differences between farmed and ‘control’ sites were detectable. No control sites were, therefore, designated or sampled.

The depths for each survey were recorded from the research vessels echo-sounder. Sampling occurred between June and September 2010. A total of seven sites, in four sea lochs (two sites each in Lochs Creran, Etive and Spelve and one site in Loch Leven) were sampled, in parallel with, but independent of, the sampling reported in Wilding [34] where further site details are given.

### Macrobenthic Sampling

Sediment samples containing macrofaunal were collected using a 0.1 m^2^ van Veen grab which was then washed through a perforated-hole 1 mm screen and the residue immediately preserved in 4% borax-buffered formalin.

Samples (total N = 108) were taken from around the mussel line sites by randomly selecting a distance along and an estimated distance off the line (based on the codes described below). The actual position of each sample was recorded from the boat’s dGPS (the aerial of which was mounted on the ‘A’ frame from which the grab was deployed). The distance-to-line for each sample was coded 1–4 based on break points of 5, 10, 20 and >20 m. More samples were collected than were to be analysed so three replicates from each distance-code, per site, were randomly chosen giving 12 samples per site (except for the sites Creran 1, Etive 1 and Leven 1 where a lack of samples at some distances meant that 10, 7 and 11 samples respectively were selected). Actual distances (i.e. not the distance code) to line, determined using a geographical information system, were used as a continuous predictor variable in subsequent statistical models with one exception: where the calculated distance was <1 m it was recoded to 1 m to reflect the sampling resolution. The total number of macrobenthic samples analysed was 76.

Macrofaunal sorting and identification was undertaken by the Fish Vet Group, Inverness, Scotland (members of the National Marine Biological Analytical Quality Control Scheme (NMBAQC) since 2006). Fauna were identified to species (except nematodes). Where species could not reliably be enumerated (e.g. colonial organisms), they were simply recorded as ‘present’ precluding their use in analyses based on counts. Univariate community metrics, consisting of the Shannon-diversity (H’), number of species and total abundance were determined using Primer (version 5, Primer-E, Plymouth, UK) [38] for each sample. In addition, the infaunal trophic index (ITI) for each sample was also calculated. ITI is an abundance weighted score that relates to differing feeding strategies with detritus feeders, interface feeders, deposit feeders and carnivores, scoring 1 to 4 respectively. Samples that are dominated by detritus feeders will have a lower score and are indicative of a degraded benthic environment [39]. ITI is routinely used as an impact metric in relation to fish-farms [40]. The univariate community metrics formed the response variables in univariate analyses (further described below).

Once fauna were removed from each sample the residue (consisting of stones, plant/algal detritus, shells and shell debris) was washed through a 12.5 mm sieve, in order to retain complete, or near complete, mussel shells. Stones, organic detritus and non-mussel shells were manually removed. The mass of the retained and drained shell material constitutes the variable ‘Hash’ which was modelled as both a response and a predictor variable (see below).

### Megabenthic Survey

A frame-mounted downward facing camera (colour, BP-L3C-High Resolution, Bowtech, Aberdeen, UK), connected by umbilical to a surface monitor and digital recorder was used to survey areas of seabed. Two 50 W lights provided the main illumination but these were augmented with two independent, parallel-mounted, torches with tightly focussed beams that were attached to the camera frame. These torches generated spots of lights that were clearly visible on the screen thus providing a datum from which the viewable area could be calculated. The camera was operated such that the light spots remained in approximately the same location giving a viewable area of approximately 1 m^2^ [35].

The camera was operated by lowering it on a winch wire till the seabed was visible. The boat and camera were then allowed to drift downstream/downwind for a period of time that varied according to oncoming obstructions (mainly moorings) and the speed of the drift. This operational approach meant the camera was directly underneath the ‘A’ frame and dGPS aerial from which the camera start and stop positions were recorded [35].

The survey start positions were dictated by the boat’s drift direction in relation to the mussel line being surveyed. Drop-down video surveys of each site consisted of a series of distance delimited contiguous transects extending to approximately 60 m from the line. The total number (across all sites) of distance-delimited surveys was 560. For the statistical analysis, each individual (distance-delimited) video survey midpoint was assigned a distance code of 1 to 6, based on the break points of 4.5, 7.5, 12, 20 and 34 m from the mussel line, and a random subset (N = 3) at each distance, stratified by Site, were selected. Therefore, for each Site there were up to 18 transects, each of which was considered a spatially independent observation. For statistical analysis the actual mid-point distance (not the distance code) was used as the predictor.

Megafauna recorded in each video transect were counted and then each was scored for the degree of mussel shell-cover: 0– shells absent, 1– occasional shells, 2– frequent shells, often touching, 3– shells covering a significant portion of viewable area but where the native seabed could be seen, and 4– shells covering the entire seabed. This ordinal scale was chosen as it separated seabed types (in relation to shell-cover) in a way that was intuitively relevant to megabenthic predators such as starfish and was relatively easy to implement. The transect area was calculated by multiplying the transect length by 1 m^2^ (viewable area) and the number of megabenthos counted. The response variable was the determined density of megafauna (starfish, see below) normalised to 10 m^2^.

### Statistical Analyses

Multivariate analyses were used to determine the relationship between macrobenthic assemblage and the amount of shell-hash per 0.1 m^2^ (continuous variable ‘Hash’) and distance to mussel line (continuous variable ‘Dist’) whilst complementary univariate analyses were used to estimate the relationship between (i) Hash versus Dist, (ii) species diversity, infaunal trophic index, number of species and number of individuals versus the predictors Hash and Dist and (iii) starfish density versus the predictors Dist and/or shell-cover. These are detailed below.

#### Multivariate analysis

Multivariate data (macrofaunal assemblages) were visualised using non-metric multiple dimensional scaling (MDS) ordination based on the Bray-Curtis similarity matrix of fourth root transformed species abundance. Fourth root transformations were used in order to down-weigh dominant species in the analysis [38]. The significance of associations between the observed patterns and the environmental drivers Dist and Hash were tested using the rank-based routine PERMANOVA [41]. In PERMANOVA the continuous variables Dist and Hash were including as co-variables in the analysis whilst Site was coded as a random variable. Non-significant terms (P>0.05) were sequentially removed from the fully-fitted model. The proportion of total variance accounted for by each term was reported in order to assess relative importance of each term.

#### Mixed modeling

The modelling of the association between the univariate response variables (e.g. community summaries and starfish density) and predictors (Dist and Hash) required mixed modelling to account for the random factor ‘Site’. Pre-analysis data exploration (checking outliers, homogeneity, normality) followed the protocol of Zuur et al (2010) [42] and, where indicated (e.g. non-linear relationships), response/predictors were log_e_ transformed with the exception of distance which was log_2_ transformed to facilitate interpretation. Where more than one predictor was modelled all were centred prior to analysis to facilitate main-effects interpretation and to reduce colinearity between predictors [43]. Model development and selection in mixed models can be relatively complex (and iterative) and the guidance given in Zuur et al (2009) [44], detailed below, was followed:

1. The beyond optimal (all fixed effects and interactions) model was initially fitted using linear regression and the residuals examined for homoscedasticity. If any residual trends were identified (versus Site or continuous predictors) a range of variance structures were tested and compared on the basis of their Akaike information criteria (AIC) score (where the lowest AIC is considered the optimal model). The goal was to identify and allow for differences in variance as a function of either categorical or continuous predictors. Residuals from the model with the lowest AIC were reassessed to check that any heteroscedasticity had been incorporated into the model.
2. The next step was to identify the optimal random component (no random effect, random intercept and random intercept and slope). Random components (intercepts and random slopes) were fitted (with the optimal variance structure, as above) and the optimal model chosen based on the lowest AIC.
3. The significance (or otherwise) of the fixed effects was then determined based on maximum likelihood (ML) parameter estimates. The full model was fitted and model terms sequentially tested using a likelihood ratio test. Insignificant terms (P>0.1) were removed. Where terms were of borderline significance (e.g. associated P value when included in the model of 0.05–0.10) then alternative models (i.e. those that retained or excluded the factor) were both reported [45].
4. Model validation was then conducted based on an analysis of residual patterns. If the residuals were normally distributed standardised residuals were plotted against predictors, including random components. Patterns in the residuals resulted in a reassessment of the model.
5. Where linear parameter estimates were not significant, or where residual patterns remained following data transformation, additive mixed models were trialled. The optimal variance structure and random components were determined as above but smoothed terms were added and their significance assessed (using the software default cross-validation setting). Non-significant smoothed terms were removed (and potentially retained as significant linear effects) and model validation was conducted as above. Final model parameters were determined using restricted maximum likelihood methods.
6. Colinearity between predictors was checked by calculation of variance inflation factors (VIFs). VIF values of <3 were considered to indicate an acceptable degree of predictor colinearity [42].

To facilitate interpretation, log transformed response variables were back-transformed to a linear scale in graphical representations. All model predictions, and 95% confidence intervals (shown graphically) relate only to the fixed factors. The variability accounted for by the random factor is given by the standard deviation of the random effects ‘intercept’ term.

All spatial data were managed using ArcGIS™ (v.9.3, ESRI, California) whilst statistical analysis was done using R [46]; mixed effect and additive mixed models were developed using the R ‘nlme’ [47] and ‘gamm’ [48] libraries respectively.

## Results

### General Site Description and Summary

**Figure 1:**
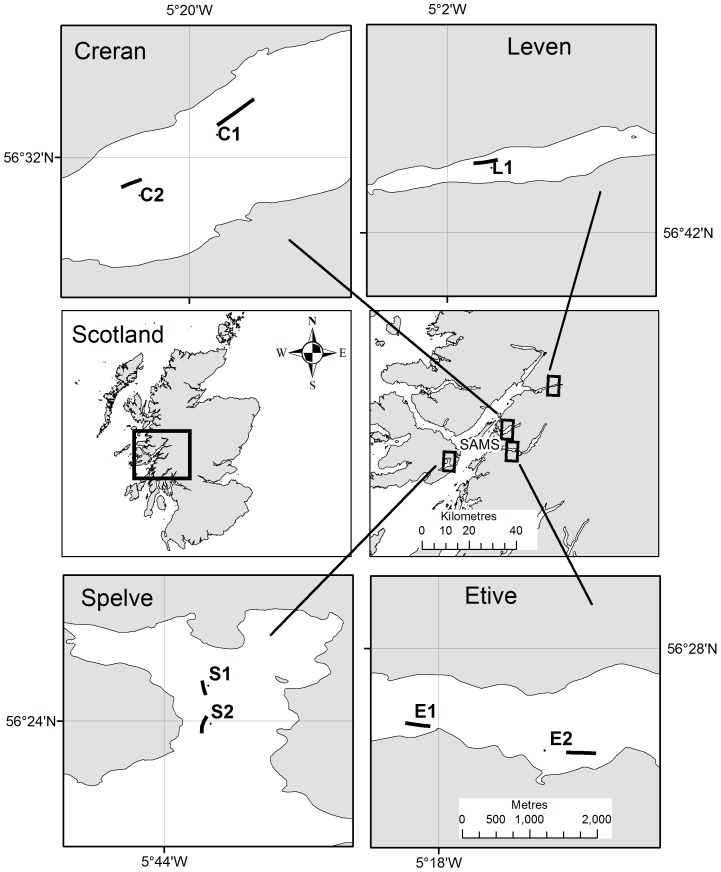
Sampling sites. Sampling sites in Creran (C1 and C2), Etive (E1 and E2), Leven (L1) and Spelve (S1 and S2). The central map (left) shows Scotland, the area within the box is shown on the right. The sampled farm sites are shown in the large-scale maps, all 1∶50,000 (scale bar shown in Etive applies to all large-scale maps). Latitude and longitude in degrees, minutes (WGS84) are shown on the larger scale maps. The location of the laboratory (‘SAMS’) is shown in the central map (right).

A total of seven sites, which hosted standing stocks of mussels, were sampled (Figure 1) with farms located in Lochs Creran, Etive, Leven and Spelve. However, active farming was only occurring in Spelve and Leven, whilst the Creran and Etive sites hosted a mussel crop that was >2 years old and which had not yet been harvested for commercial reasons. A summary of the sediment characteristics at each Site is given in Wilding [34], further Site descriptions (based on DDV surveys) are given here. The epibenthic megafauna at the Creran 1 and 2 sites were characterised by starfish (see below) and the urchins *Psammechinus miliaris* Forbes with occasional seapens (*Virgularia mirabilis* (Muller, 1776)). At the Creran sites the seabed adjacent to the lines frequently contained black patches where crabs or other bioturbators had disturbed the surface which was frequently mottled dark brown in appearance. These areas commonly contained numerous detached epifouling organisms (notably the sea squirt *Ciona intestinalis* (L.)) considered likely to have originated on the mussel lines. The Etive 1 site hosted large numbers of the sea cucumber *Psolus phantapus* (Strussenfelt, 1765) (NW section) whilst the ophiuroid *Ophiura ophiura* (L.) was commonly observed on level sediments together with occasional patches of the seapen *V. mirabilis* at Etive 2. At the Leven site large numbers of the anemone *Protanthea simplex* Carlgren, 1891 were observed growing on rope-moorings/hard benthic substrata. A bed of the brittle star *Amphiura filiformis* Muller 1776, was observed within 10 m of the north-east part of the Leven farm site. At the Spelve 1 and 2 sites the urchin *P. miliaris* was commonly observed and, in shallower areas, benthic diatoms were common (as indicated by a uniform brown/green covering) through which tracks of the whelk *Buccinium undatum L*. were frequently observed.

### Macrobenthic Results

#### General observations

Sediment sampling using a grab was straightforward around mussel farms, even in very close proximity to the lines, with a heavy duty 0.1 m^2^ grab penetrating and closing around the relatively soft shell-hash well. Some samples consisted almost entirely of shell-hash and these were characteristically black and frequently smelled of hydrogen sulphide, a phenomenon particularly noticeable at the Leven 1 site where terrestrial detritus was also commonly observed. At both Creran sites, some samples from adjacent to the mussel line also smelled strongly of hydrogen sulphide but these were not necessarily associated with high levels of Hash. Unoccupied shells of *Turritella sp*. were common at Creran 1 and Spelve 1 whilst terrestrial detritus was also common at Creran 2. Samples from Etive 1 and 2 were noticeably coarser compared with the other samples and contained fine grit, numerous rocks and stones in addition to shell-hash and varying amounts of unidentified algal fragments.

Overall, the dominant macrobenthic species associated with close proximity to mussel lines was highly variable but frequently included species that are indicative of at least a moderate degree of organic enrichment. The organic-enrichment indicator polychaete *Capitella* sp was the most numerically dominant species at both the Creran sites, in closest proximity to the line (<5 m, mean 90 and 45 individuals per grab (IPG) at Site 1 and 2 respectively). All stations at Etive 1 were dominated (30–80 IPG) by the polychaete *Chaetozone zetlandica* McIntosh, 1911 (the genus being associated with organic enrichment [49]) but this species was largely absent from Etive 2 where the polychaete *Sige fusigera* Malmgren, 1865 was most abundant nearest the line (24 IPG). The near-line (<5 m) samples from Loch Leven were dominated (∼200 and 40 IPG respectively) by the polychaete *Protodorvillea kefersteini* (McIntosh, 1869) and annelid *Tubificoides benedii* (Udekem, 1855) both of which are indicators of moderate organic enrichment [50] and these were also present, but less abundant, at stations >5 m from the line. Sediments near (<10 m) to Spelve, Site 1, were dominated (∼210 IPG) by the opportunistic, surface deposit feeding polychaete *Aphelochaeta* sp Blake, 1991 [51] contrasting with Spelve, Site 2 where the bivalve *Kurtiella bidentata* (Montagu, 1803) was the most numerous of the macrobenthos identified.

### Multivariate Patterns and Associations

**Figure 2:**
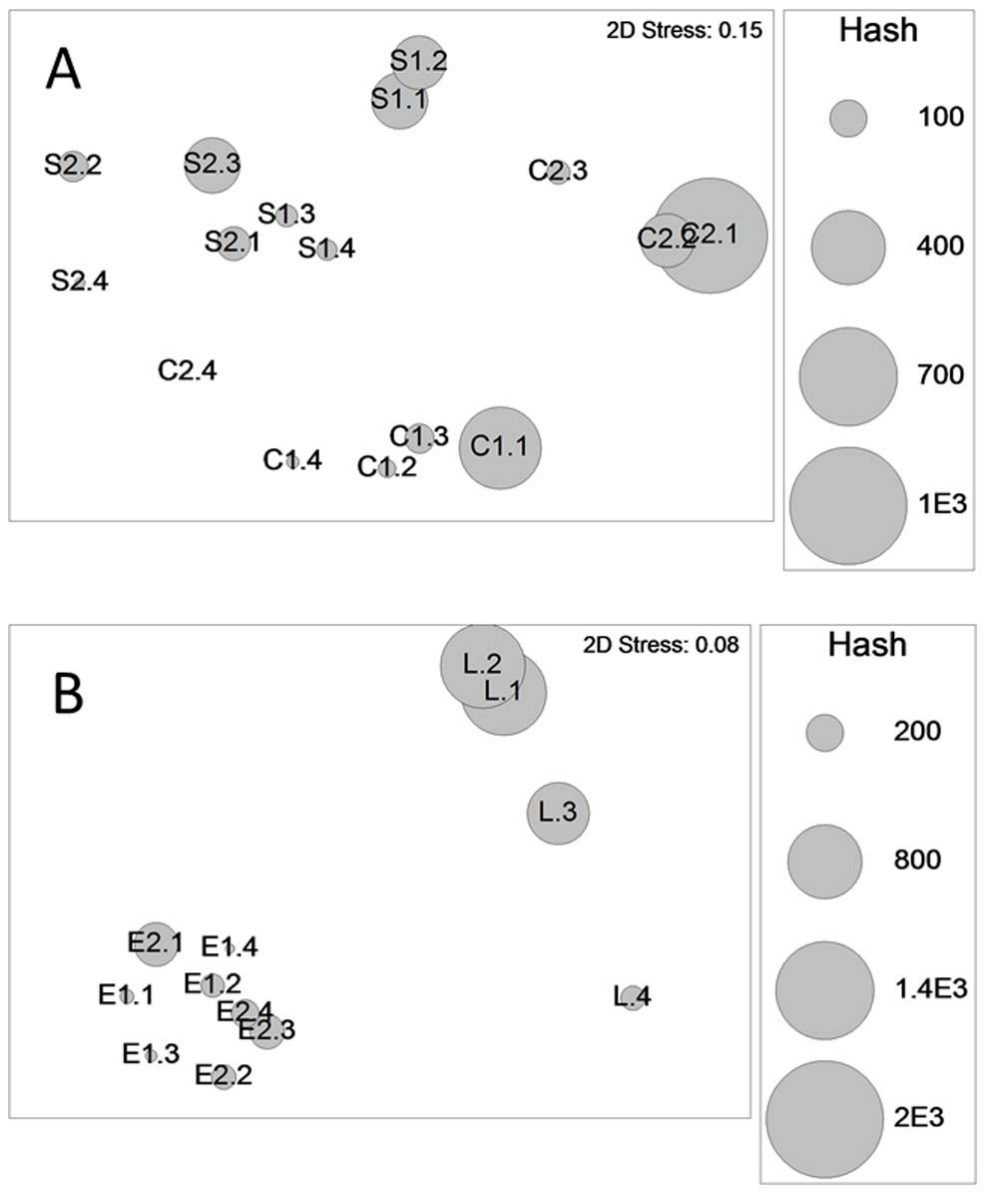
MDS relationship between assemblage, loch, distance-code and shell-hash content (bubble size). Note: a single ordination was first obtained which split the Sites into two distinct groups: (A) Creran and Spelve and (B) Etive and Leven which were analysed as MDS subsets. Bubble size indicates hash content (in g per grab), Code: C – Creran, E – Etive, L – Leven, S – Spelve, 1^st^ number is the site (e.g. C1 is Creran 1 except for Leven where there was only one site), the second number is the distance code (1 is the closest to the mussel line; see methods). Note that the bubble scaling differs between the figures.

There were clear differences in the overall species assemblages between sites (PERMANOVA, Factor Site, Table 1). These differences are clearly shown in the MDS (Figure 2 A and B) which split the sites into two main groups (Creran & Spelve versus Etive & Leven respectively). However, within these broad locational differences, there was also evidence of changes in assemblage associated with Dist and Hash.

**Table 1:** Results from the PERMANOVA analysis (based on the Bray-Curtis similarity distances of fourth-root transformed species abundance).

Source	df	MS	Pseudo-F	P(perm)	% variance
Dist	1	5738.4	2.83	0.0002	6.0
Hash	1	11404	2.26	0.0012	9.0
Site	6	11217	9.08	0.0001	27
Dist×Hash	1	2909.9	2.35	0.0026	4.2
Dist×Site	6	2566.2	2.08	0.0001	10
Hash×Site	6	2022.6	1.64	0.0027	12
Residual	5	1235.9			31

df- degrees of freedom, MS – mean square, P(perm) - rank-based permutational based probability, % variance component indicates relative importance of each term. Dist – distance. The Dist:Hash:Site interaction was not included as it did not make a significant improvement in model fit.

PERMANOVA showed that the effect of both Dist and Hash on assemblage structure depended on Site (Table 1). The MDS (Figure 2 A and B) allows interpretation of this dependency: the relationship with Hash was most pronounced in Lochs Creran and Spelve (grabs containing similar Hash are grouped together) whilst the Dist association was most clearly shown in Loch Leven (Figure 2 B). In Lochs Creran and Spelve the community structure was similar between those samples located within 7 m of the mussel line (samples C1– C3 and S1– S3 respectively, Figure 2 A) but quite distinct from those located >20 m from the line (C4 and S4, Figure 2 A). This did not apply to Loch Etive where the major assemblage difference occurred between samples of <3 m from the line and all others which were broadly similar (and where Hash was present in all samples).

### Univariate Analysis

#### Relationship between distance and sediment shell-hash content

Sediment Hash content, per Site, was heavily right-skewed with occasional samples containing large amounts of Hash (compare mean and median values e.g. Creran 1, Table 2) indicating a high-degree of patchiness. There was considerable variability in Hash between Sites, reflected in the standard deviation multipliers in the weighted regression which ranged between 0.230 and 1.05 (Table 3).

**Table 2:** Hash content at the seven sampled sites.

Sites	Median	IQR	Mean	Sd	Minimum	Maximum	N
Creran 1	23	50	226	602	0	2116	12
Creran 2	10	62	264	610	0	1920	10
Etive 1	28	38	46	40	12	126	7
Etive 2	121	128	144	104	6	316	12
Leven 1	756	777	651	443	0	1163	12
Spelve 1	97	164	126	115	19	371	12
Spelve 2	52	88	85	126	3	445	11

IQR – interquartile range, Std - standard deviation, all values are g per 0.1 m^2^ grab except N (sample size).

**Table 3:** Log Hash (g per grab) as a function of log_2_ distance (m): results from general additive mixed modelling (GAMM), weighted by Site (random factor).

Random effects: Site			Intercept				Residual						
Standard deviation			0.831				1.81						
**Fixed effects**			LogHash ∼ s (Log_2_Dist)										
			Estimate		Std.error			T value		Pr (> T)			
Intercept			3.95		0.351			11.29		<2×10^−16^			
			Estimated df					F value		P value			
Smooth term			2.33					25.4		<2×10^−9^			
Weightings (estimated relative standard deviation per Site)													
Site	Creran 1	Creran 2		Etive 1		Etive 2			Leven 1		Spelve 1	Spelve 2	
	1.00	1.05		0.230		0.708			0.901		0.343	0.543	

**Figure 3:**
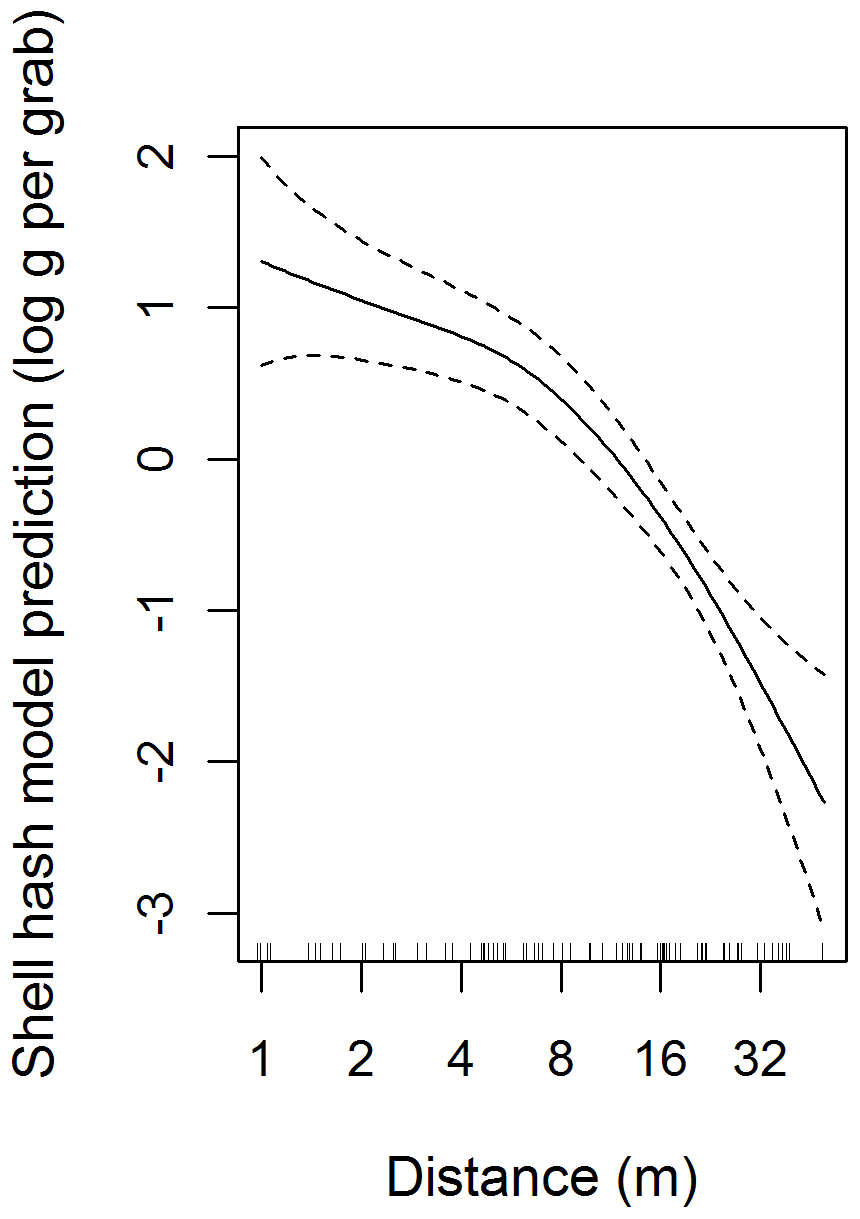
GAMM model illustrating shell-hash (Hash) as a function of distance (Dist). The rug on the bottom axis illustrates sample positions. The solid line indicates the model predictions with 95% confidence intervals shown as dashed lines.

Overall, hash content decreased as a function of distance from the line but, on a log_2_ scale, the nature of the relationship was non-linear as indicated by the significant smooth term (Table 3). The optimal GAMM was based on a random intercept indicating there was no significant advantage in modelling the relationship between LogHash and Log_2_Dist separately for each site. The GAMM predicted relatively high shell-hash contents in sediments until a distance of approximately 5 m whereafter it declined rapidly (Figure 3). The 95% confidence intervals (Figure 3) indicate that, at a distance of 1 m from the line, Hash would be expected to be present between 1.65 and 6.05 times the mean amount (for any site) and that this would decrease at a distance of approximately 32 m to 0.135–0.368 times the mean content representing a 5–45 fold decrease in content over this distance.

#### Relationship between Univariate Assemblage Measures, Dist and Hash

Four univariate measures of environmental status were considered (Shannon diversity (H’), number of species, total abundance and infaunal trophic index (see methods) and only total abundance was statistically significantly linked to the predictors. Inspection of several models linking total abundance and the predictors Dist and/or Hash identified two differing, but plausible, alternatives (Model 1 and Model 2; loglikelihood ratio between the models was 5.50, P = 0.0640). The optimal models (Model 1 and 2) both contained the random intercept term for Site indicating that abundances varied significantly between sites but differed in terms of their fixed effects (see below). The standard deviations for the random intercept term and residual in both models were approximately equal (Table 4, Table 5). However, of the random effects, the residual term was associated with the larger standard deviation indicating that these data were characterised by a large degree of noise that could not be accounted for by the model (Table 4, Table 5).

**Table 4:** Model 1: Log macrofaunal abundance as a function of Log_2_Dist * LogHash.

Random effects: Site			
	Intercept	Residual	
Standard deviation	0.297	0.407	
**Fixed effects**			
Factor	Estimate	std. error	P
Intercept	5.25	0.124	<0.001
Log_2_Dist	−0.133	0.0392	0.00120
LogHash	0.0433	0.0328	0.191
Log_2_Dist*LogHash	−0.0326	0.0155	0.0396

Site was modelled as a random intercept. The predictors were centred prior to analysis.

**Table 5:** Model 2: Log macrofaunal abundance as a function of Log_2_Dist.

Random effects: Site			
	Intercept	Residual	
Standard deviation	0.257	0.418	
**Fixed effects**			
Factor	Estimate	std. error	P
Intercept	5.79	0.156	<0.0001
Log_2_Dist	−0.160	0.0324	<0.0001

Site was modelled as a random intercept.

**Figure 4:**
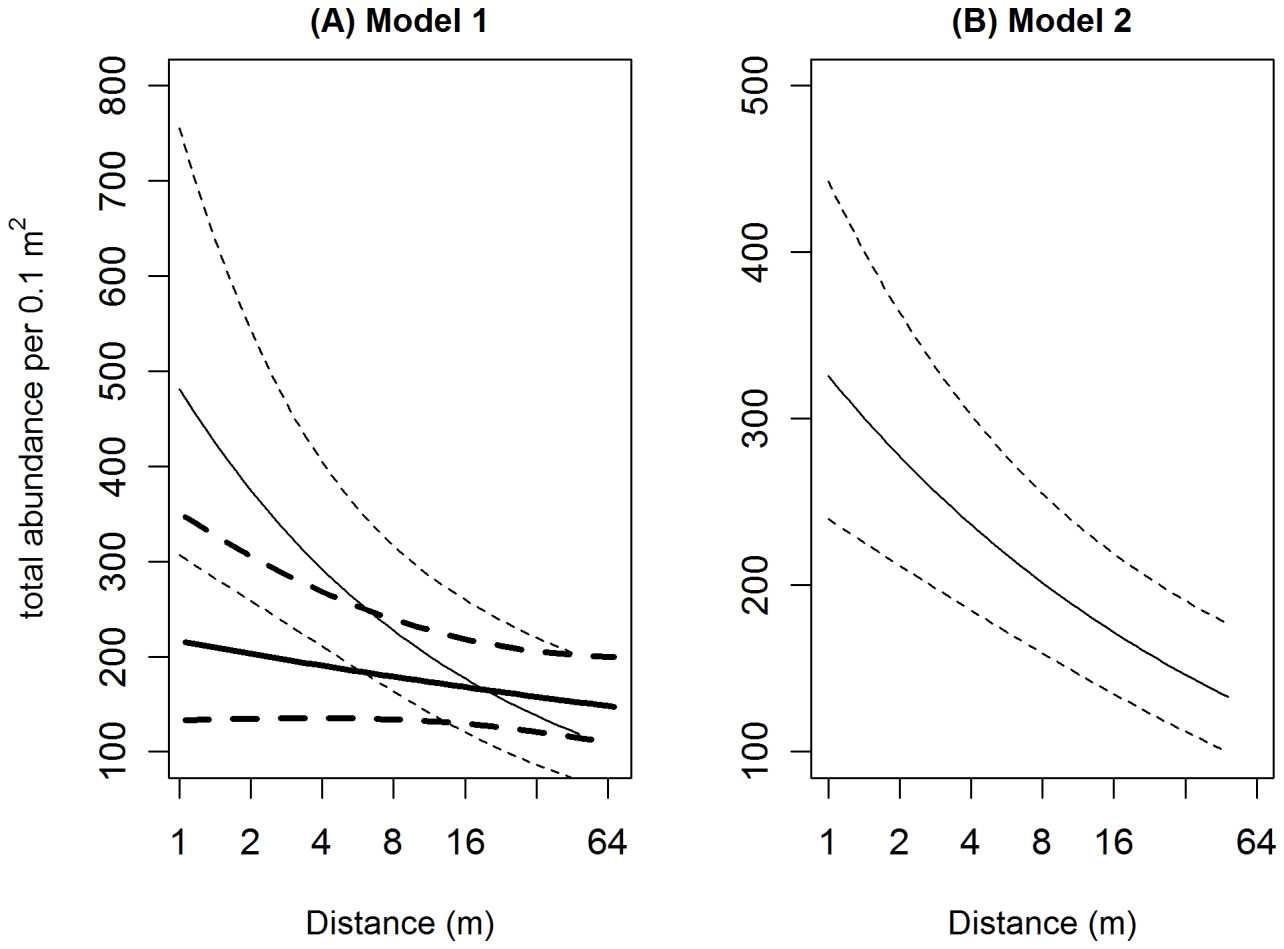
Total macrofaunal abundance predictions from alternative models. (A) Model 1: relationship between total faunal abundance and Distance (log_2_ scale) at 1000 g per hash per grab (light lines) and 50 g hash per grab (heavy lines) respectively. Model 2 (B) total abundance as a function of distance. The total abundance has been back transformed from the log_e_ scale used in the model. For both models, the solid line indicates the model predictions with 95% confidence intervals shown as dashed lines.

Model 1 and Model 2 are interpreted separately here. Model 1: macrofaunal abundance was negatively associated with Log_2_Dist but this effect was influenced by the amount of shell-hash present (Log_2_Dist: LogHash interaction, Table 4) such that the increase in macrofaunal abundance, in relation to line proximity, was more pronounced in the presence of high levels of shell hash (e.g. 1000 g per grab, Figure 4 (A)). At high levels of shell-hash macrofauna were between approximately 1.5 and 8.0 times as abundant adjacent (∼1 m) to the mussel line compared with 64 m distance. At average levels of shell-hash (50 g per grab and setting centred LogHash = zero in Table 4) Model 1 indicates that macrofauna could be expected to decline by a factor of 0.88 (95% CI 0.81, 0.95) for each doubling of distance equating to an approximate halving (95% CI 0.28, 0.70) of macrofaunal abundance over the range 1–64 m.

Model 2 - this more parsimonious model, which excludes LogHash as a factor, indicated a linear relationship between decreasing macrofaunal abundance (log transformed) and increasing Log_2_Dist where macrofaunal abundance decreased by a factor of 0.862 for each doubling of Dist (Table 5). Model 2 predicts that, at an average site, macrofaunal abundance will decline from approximately 325 (95% CI 245, 450) to 130 (95% CI 100, 175) per 0.1 m^2^ grab over the distance 1–64 m from the mussel line (Figure 4 (B)).

### Megabenthic Survey Results

Initial assessment of video from all sites indicated considerable differences in substratum colour, particularly in relation to the amount of surficial shell-hash. On certain dark backgrounds, counting cryptic species was difficult potentially confounding the results. For these reasons, only members of the ubiquitous predatory/scavenging class Asteroidea (starfish including *Asterias rubens* L. and *Henricia* sp. Gray, 1840), which were easily identified on any background and relatively common at all sites, were counted.

**Figure 5:**
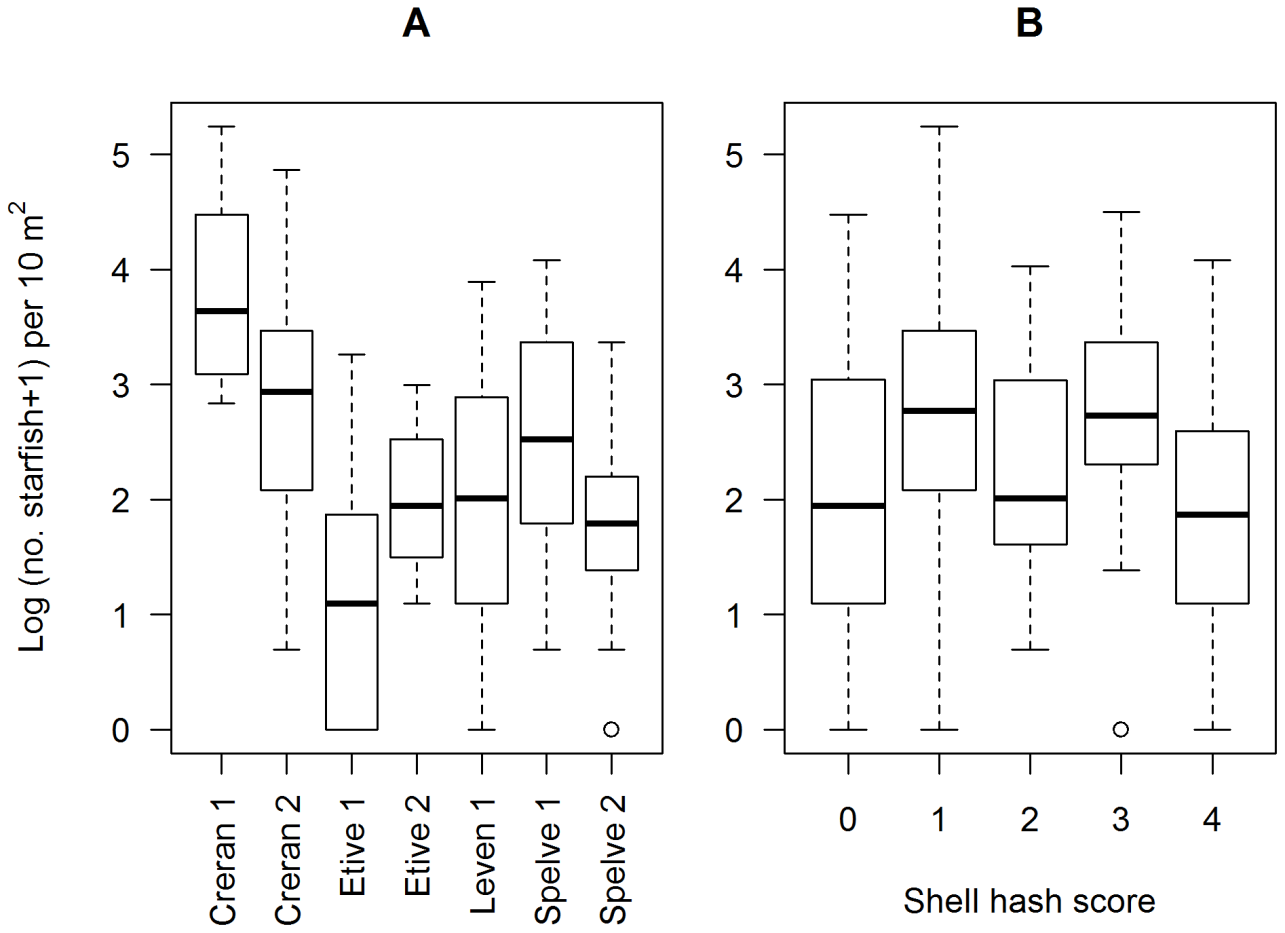
The relationship between log (number of starfish +1) per 10 m2 in different lochs (A) and compared with shell-hash score (B). The median abundance is indicated by the horizontal line within the box (interquartile range), upper and lower serifs represent the upper and lower adjacent values.

Starfish abundance ranged widely within and between sites. Maxima ranged between 19 and 188 per 10 m^2^ (Etive 2 and Creran 1 respectively, Figure 5 A). The shell cover score ranged between 0 and 4 for all sites with the exception of Creran 1 where the highest score was 3. There was no consistent linear relationship detected between shell cover score and starfish abundance (Figure 5 B).

**Figure 6:**
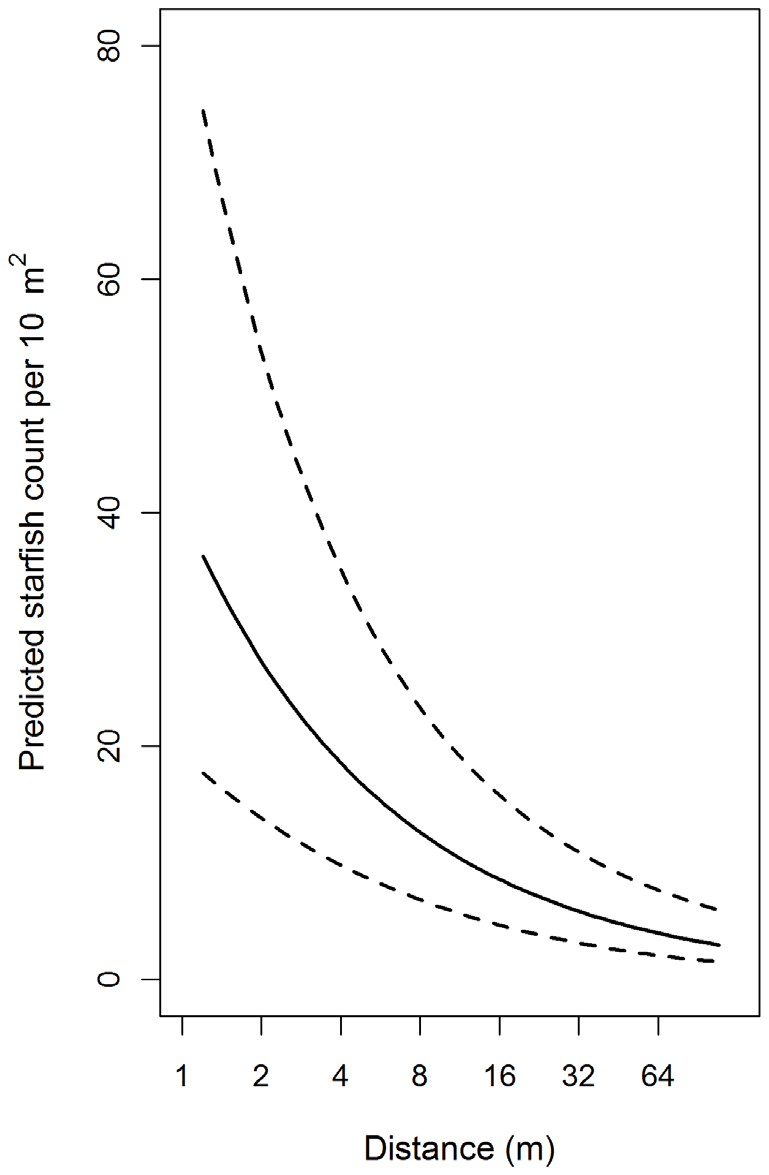
Mixed model predicted (solid line) numbers of starfish v. distance. Predictions are back-transformed. The dashed line represents the 95% confidence interval.

Whilst the abundance of starfish varied between sites (the model containing the random intercept term was superior) there was no evidence that the relationship between distance and abundance changed between sites (i.e. the slope coefficient was constant between sites). However, the number of starfish per site was highly variable (standard deviation multipliers ranged between 0.848 and 1.38, Table 6). The density of starfish decreased by a factor of 0.68 for every doubling of distance from the mussel line (Table 6). On average (across Sites) starfish densities could be expected to decrease from 40 (95% CI 20, 80) at 1 m to 4.0 (95% CI 3.0, 10) per 10 m^2^ at 64 m (Figure 6) an approximate 2.0–27 fold decrease.

**Table 6:** Starfish density as a function of distance weighted by Site which was also modelled as a random intercept.

Random effects: Site						
		Intercept		Residual		
Standard deviation		0.797		0.771		
**Fixed effects**						
Factor		Estimate		std. error	P	
Intercept		3.69		0.375	<0.001	
Log_2_Dist		−0.386		0.0564	<0.001	
Weightings (estimated relative standard deviation per Site)						
Creran1	Creran 2	Etive 1	Etive 2	Leven 1	Spelve 1	Spelve 2
1.00	1.29	1.38	0.848	1.29	0.888	0.914

## Discussion

Mussel farms on the west coast of Scotland were responsible for several changes in the sediment and associated macro- and megabenthic fauna around them. The most visually apparent change was in the amount of shell-hash present which frequently dominated the sediment in close proximity to the mussel lines. The presence of shell-hash and distance from the mussel line both influenced the macrobenthic assemblage structure but there was no evidence of a commensurate change in macrobenthic diversity or feeding strategy (as revealed by ITI). Mussel farm proximity and/or shell-hash was associated with an increase in total macrobenthos but only proximity was linked to starfish density.

### Methodology

The research reported here is one of the most comprehensive cross-sectional studies examining the extent of macro–and megabenthic changes occurring around mussel farms. The sampling techniques used worked well: the van-Veen grab was able to sample effectively even in the presence of very high levels of shell-hash and, in this way was superior to sampling with a coring device [34], whilst the drop-and-drift video approach resulted in excellent coverage around mussel farms [35]. Working in very close proximity (∼1 m) to the mussel lines was relatively straightforward with both techniques in a relatively calm protected sealoch environment and the spatial resolution, from the ‘A’ frame mounted dGPS aerial, was excellent as no offset (lay-back) calculations were necessary to georeference the sample locations (including images - compared with towed video or remotely operated vehicle imaging). These techniques are recommended for sampling around mussel farms in sheltered environments.

Any observational programme is limited through the logistics of site access and sampling time/cost. In the current case, spatial coverage was limited to approximately 50 km of the laboratory and the cost of macrobenthic sorting and identification limited the macrobenthic sample size. The determination of shell-hash per grab using the techniques described was straightforward, rapid and cost-effective. Drop-down video was also rapid and cost-effective but, as with any underwater visualisation technique, was potentially limited (though not biased) as a function of water clarity and the motion of the survey vessel (discussed in [35]). The ability to identify cryptic megabenthic species will vary according to the substratum type, for example, the urchin *P. miliaris* was relatively easy to observe and identify on a plain, light brown background, but very difficult to identify when located on dark heterogeneous backgrounds such as shell-debris hence the decision to count only starfish.

### Shell-hash Accumulation around Mussel Farms

Living mussels are dense and will sink relatively rapidly to the seabed limiting their lateral dispersion as they fall through the water column. That the apparent hash field extended with little attenuation until 8 m then declined rapidly may be explained by the lateral movement of the mussel line in response to wind/tide or storms rather than the horizontal advection of living or dead shells. These results cannot indicate the accumulation rate of mussel hash but the observation that some grabs consisted mostly of shell-hash (>2 kg hash per grab) together with visual observations (hash score of 4 commonly observed) shows that hash was frequently a dominant feature in soft-sediments around mussel lines. This is in agreement with Mattsson and Linden [29] who reported accumulations of 3000+/−1000 shells m^−2^ year^−1^, Harstein’s [18] ‘facies A2’ which appears to be a 100% cover and the 50% seabed coverage reported by Inglis et al [19]. The implications of these accumulations are discussed below.

### Macrofaunal Changes around Mussel Farms

The macrobenthic community within the sampled areas varied considerably, with Site being the most important determinant of assemblage structure (as revealed by PERMANOVA). However, samples taken from near the line were characteristically dominated by species which were indicative of a degree of organic enrichment (e.g. *Capitella* sp. at the Creran sites). Within Sites, proximity to mussel line and the amount of hash in the sediment both influenced assemblage composition. Within most Sites, samples containing similar proportions of shell-hash hosted similar assemblages, and these samples tended to be nearer the mussel line (as above). However, the multivariate analyses showed that whilst the shell-hash effect on assemblage structure depended on distance, hash and distance also had independent effects and that, in isolation, shell-hash had a greater influence, compared to distance, as a driver of assemblage structure.

The univariate responses species diversity, species richness and ITI were not clearly related to the predictors distance and hash. This is potentially contrary to the results of the multivariate analyses (above) but may be explained in two ways. Firstly, multivariate techniques are more powerful (less liable to Type II errors) compared with their univariate counterparts and this study may not been of sufficient scope (sample size) to accurately estimate small changes in univariate measures that were occurring. Alternatively, the changes in species in response to increasing Dist (species increasing along an oxygen concentration gradient, see [34]) and Hash (species decreasing in response to decreasing habitat heterogeneity related to mussel debris) may have countered each other resulting in limited change in univariate measures such as diversity (or richness/ITI) even where more substantial changes in species structure were occurring.

Of the univariate metrics examined, the overall abundance was most clearly related to the predictors Dist and Hash. Mixed model selection can be subjective and two plausible models are presented here. The multivariate analysis indicated that shell-hash was a factor in determining assemblage composition hence it is reasonable to include it in the model. The two models both showed a negative relationship between distance and macrofaunal abundance but, according to Model 1, the effect of distance depended on the amount of shell-hash in the sediment. It is plausible the surface roughness caused by surficial hash will act to increase the accumulation of organic material [20], as has been reported for maerl in relation to fish-farm detritus [21]. Interpretation of these data (Model 1) is commensurate with the observations of Hartstein and Rowden [52] and Grant et al [53] who both reported that mussel debris was a good predictor of benthic impact.

The results presented here should be interpreted in conjunction with the findings of Wilding [34] which, at the same sites, reported a non-linear relationship between redox (a proxy for sediment oxygenation and potential driver of macrobenthic assemblage) and distance. The major decrease in redox recorded by Wilding [34] (Figure 4) extended to approximately 8 m and increased thereafter The redox-distance relationship corresponds (inversely) with the hash field–distance relationship recorded here which also extended to approximately 8 m and decreased thereafter. Together Wilding [34] and the data presented here provide good evidence for a role of shell-hash facilitating a moderate accumulation of organic matter (e.g. through enhanced particle trapping, see correlation between carbonate and organic carbon content, Figure 3 in Wilding [34]) and that this drives changes in the macrobenthos (reported here). Variability in the relationship between the response and hash might be because the model could not distinguish surficial- and entrained-hash and these may have different effects on benthic infaunal assemblages and related sediment processes.

The alternative (more parsimonious) model (Model 2) indicated a simple reduction in macrofaunal abundance as a function of distance. This is commensurate with the findings of Stenton-Dozey et al. [33] who also found total biomass a good indicator of benthic impacts around mussel farms (Saldhana Bay, South Africa). The total macrofaunal abundance reported here, adjacent to the mussel cage edge (∼3000 individuals m^−2^) corresponds to the abundance predicted at a distance of ∼ 25 m from Scottish fish-cages [54]. Differences in the distance-abundance relationship between fish-and mussel-farms are likely to be a consequence of differences in organic loading. There was no evidence of a reduction in benthic diversity occurring around mussel-farms and this contrasts with fish-farms even when comparing sediment at distances subject to similar organic loading (e.g. comparing mussel-farm edge and 25 m fish-farm distance). The differences in biodiversity impacts may be as a consequence of differing aquaculture practices, for example the use of chemotheraputants and/or agents to control net-fouling on Scottish fish-farms [55].

### Benthic Scavenger Changes around Mussel Lines

Any biological monitoring programme should monitor species that are relevant to the impact source and that are easily identified, common and ubiquitous [56]. In relation to the megabenthos around the sampled mussel farms, starfish (class Asteroidea) met all these criteria.

Starfish, and other benthic scavengers/predators, are attracted to living mussels lying on the seabed [20], [57], [58] and these, and other detached epibiota, are likely to be closely associated with the mussel line. The data presented here indicate that starfish density can be expected to decrease by 1.47 times for every doubling of distance from the mussel line and that there were 2–27 times more starfish in close proximity (1 m) to the line compared with stations ∼ 64 m from the line. Other research has indicated comparable results, for example Inglis and Gust [19] found starfish to be up to 39 times more abundant under mussel farms compared with control locations. Whilst there was no evidence that starfish were associated with shell-cover they were patchily distributed (in concurrence with D’Amours et al. [36]). This is commensurate with their attraction to short-term mussel-line derived food which they quickly consume leaving empty shells that subsequently remain on the seabed. The fate and behaviour (e.g. longevity) of this shell-hash material is not currently understood.

### Management Implications

Any intervention in the marine environment (of any scale) will have an effect on all relevant response variables (e.g. diversity) [56], and this will, theoretically, extend infinitely far (based on a multiplicative relationship between impact and distance). Whether this is ‘detected’ as statistically significant will depend on, principally, the effect size/variance ratio and the sample size [56]. Environmental managers charged with limiting environmental damage need to justify threshold values beyond which change (in a relevant response) is unacceptable [56]. With this caveat in mind, the data presented here, and in Wilding [34], support the hypothesis that whilst mussel farms (within the sampled population) were responsible for a degree of shell-hash linked organic enrichment, it was of insufficient magnitude to effect broad-scale, major sedimentary changes that are typically linked to reductions in macrobenthic diversity, at least beyond the farm boundary. The only detectable overall change was a localised decrease in redox [34] and a concomitant increase in faunal abundance (reported here) indicative of mild organic enrichment. This is in contrast with other forms of aquaculture e.g. fish-farming, where the sediments adjacent to the farm are characterised by a heavily modified, specialist assemblage [23] but, as with fish-farm impacts [22], the results here showed a high-degree of site-specific variability. In terms of soft-sediment benthic impacts, and the metrics measured, the mussel farms represented by those sampled here can be considered a relatively benign method of producing food.

Wong et al (2012) [27] suggested that the mapping of shell-hash would be a reasonable indicator of the ‘limit’ of mussel farm impacts. The shell-hash monitoring technique developed here would allow cost-effective, real-time, mapping of the shell-hash field around mussel farms and offers a useful monitoring tool. The data presented here suggest that detecting mussel-farm impacts, related to macrobenthos and starfish, beyond 10 m from the farm periphery will be challenging where they are located on soft sediments (i.e. would require a greater number of samples than reported here to find statistically significant differences). For logistical reasons this research focussed on mussel-farm soft-sediment interactions. However, many mussel farms, particularly those close to the shore and over steeply inclined seabeds, overlap hard-substrata. Research into the impact of mussel farms on these habitats is urgently required.
